# The Story of the Insane from Year to Year

**Published:** 1900-04-14

**Authors:** 


### THE STORY OF THE INSANE FROM YEAR TO YEAR.

Twelfth Series.

### THE WEST SUSSEX ASYLUM AT CHICHESTER.

The number of patients in this asylum at the beginning of the year 1898 was 430, and at the end of the year it had increased to 466. There were 94 first admissions, 26 re-admissions, 24 recoveries, and 30 deaths. The recoveries stand at 22·93 per cent. of the admissions, and the deaths at 6·63 per cent. of the average number resident. Both of these percentages are considerably below the County Asylum average. Of the deaths during the year 14 were caused by cerebral and spinal diseases, 5 by consumption, and 1 by enteritis. It is curious that this disease should have made its appearance in an asylum not two years old, but we do not notice any reference to the supposed cause, nor whether the disease was limited to the fatal case. Of the year's admissions 35 owed their insanity to various moral causes, 24 to intemperance in drink, 52 to hereditary influence, and 11 to influenza. As to the latter Dr. Kidd remarks that it must now take its place "as a recognised factor in the ætiology of insanity." Examining the admissions, Dr. Kidd can only find 29 in which recovery is probable, and 17 in which it is possible, so that it cannot be a matter of surprise that the recovery rate is low. Post-mortem examinations were made in every case of death, a state of things which we regret to notice, and hope that a more enlightened practice will prevail in future. The medical officers have delivered lectures to the staff, and 40 nurses and attendants entered for the examination of the St. John Ambulance Association. Of these candidates 33 passed—a very fair proportion. As already stated, the asylum is not two years old, and yet additions for 300 patients are to be commenced. That these are necessary we fully believe, but why the asylum was not made large enough to begin with is a question that might very reasonably be asked. The committee say that "Dr. Kidd continues to carry out the responsible duties of his office in the same efficient manner as hitherto." The weekly rate of maintenance is 12s. 3d. a head. This will fall as the asylum gets older.

### THE WESTERN COUNTIES ASYLUM FOR IDIOTS, EXETER.

This institution contained 250 patients at the end of the year 1898. There were 33 admissions, 3 recoveries, 8 much improved on discharge, and 16 moderately improved. In spite of the unpromising material of which the members in idiot asylums is made up, we learn that three of the patients discharged are now earning their own living, and that four are capable of contributing towards their support. The financial position of the asylum is good. Nearly £1,800 of the building debt has been paid off during the year, and only £121 remained unpaid. The total income was £7,500. Of this £7,159 was derived from the maintenance rates charged for patients, and the rest from annual subscriptions, donations, dividends, and sale of children's work. The asylum is under lay management, but there is also a resident medical officer.

### THE MULLINGAR DISTRICT ASYLUM.

The limits of accommodation are stated to be for 696 patients; but there were 742 in residence at the beginning of the year and 772 at the close of it. There were 111 first admissions, 37 readmissions, 56 recoveries, and 45 deaths. The recoveries stand at 37·8 per cent. of the admissions, and the deaths at 5·8 per cent. of the average number resident. Of the deaths 11 were caused by consumption, 2 by pneumonia, and 5 by other pulmonary diseases. Of the year's admissions 10 owed their insanity to moral causes, 9 to intemperance in drink, and 40 to hereditary influence. Dr. Finegan says that a case of fever occurred in the asylum which had all the symptoms and post-mortem appearances of typhoid, but in which, nevertheless, the bacillus was not to be found. Other writers have pointed out that there is a disease clinically identical with typhoid, but which exists in sporadic cases only, and it was probably one of these that Dr. Finegan had under treatment. The Irish asylums have had to pass through a crisis similar to that of the English ones when the County Councils came into power. We hope it may be as pleasantly effected in the sister Isle as it was with us; but we can all appreciate the feelings which Dr. Finegan expresses in the closing sentences of his report. The average cost per head per annum was £28 4s.

